# The effects of peripherally-subacute treatment with irisin on hippocampal dendritogenesis and astrocyte-secreted factors

**DOI:** 10.20463/jenb.2019.0029

**Published:** 2019-12-31

**Authors:** Mun-Hee Kim, Yea-Hyun Leem

**Affiliations:** 1 Korea National Sport University, Seoul Republic of Korea; 2 Department of Molecular Medicine, Ewha Womans University, Seoul Republic of Korea

**Keywords:** FNDC5/irisin, dendritic remodeling, hippocampus, hevin, TGF-β1

## Abstract

**[Purpose]:**

Fibronectin type III domain containing 5 (FNDC5)/irisin is an exercise-induced myokine, which contributes to cognitive functions. However, the relationship between the neuroprotective effects of FNDC5/irisin and hippocampal dendritic remodeling and astrocyte-secreted factors remains unclear. Therefore, we explored whether subchronic recombinant irisin treatment affected hippocampal morphology and some astrocyte-derived molecules.

**[Methods]:**

Mice were intraperitoneally injected with irisin (0.5 μg/kg/day) for seven days, followed by their sacrifice two days later. Hippocampal morphometric parameters were analyzed and *pgc-1a*, *fndc5*, *bdnf*, and some astrocyte-derived factors mRNA levels were measured.

**[Results]:**

Dendritic length, arborization, and spine density were enhanced by irisin regimen in hippocampal CA1 and CA3 areas. Hippocampal *pgc-1a*, *fndc5*, and *bdnf* mRNA levels were significantly increased by irisin treatment. Moreover, *hevin* mRNA levels were significantly enhanced, whereas *tgf-b1* levels downregulated by irisin treatment.

**[Conclusion]:**

FNDC5/irisin has dendritogenic activity probably through hevin induction and TGF-β1 suppression.

## INTRODUCTION

Irisin, which is cleaved from fibronectin type III domain containing 5 (FNDC5), is an exercise-responded myokine under controlling peroxisome proliferator-activated receptor-γ coactivator 1α (PGC-1α)^1^. Exercise-induced circulating irisin can cross the blood-brain barrier (BBB) and then induce the brain-derived neurotrophic factor (BDNF) in the hippocampus^2^. Mounting evidence has described the synaptic plasticity-promoting role of irisin in memory processes under physiological and pathological conditions such as Alzheimer’s disease (AD)^2-3^.

Synaptic plasticity is a crucial mechanism underlying memory processes, in which especially the structural plasticity such as dendritic rearrangement plays a regulatory role in synaptic connectivity. Astrocytes, which are the most abundant glial cells in the brain, provide crucial metabolic and trophic support to neurons^4^. Astrocytes of the gray matter are structural and functional components of synapses, which secrete diverse synaptogenic signals to control synaptic formation and function, including thrombospondins (TSPs), hevin, secreted protein acidic and rich in cysteine (SPARC), and transforming growth factor β-1 (TGF-β1)^5-8^. The astrocyte-secreted factors have been well-studied in a purified retinal ganglion cell (RGC) culture system, in which TSPs and hevin induce the excitatory synapse formation by binding to α2δ-1 and bridging between Neurexin-1α and neuroligin-1B, respectively^5,9^. Moreover, in the dentate gyrus area, the decrease occurred in proliferating cells but not in differentiating cells in SPARC KO mice^10^. TGF-β1 contributes to the formation of both excitatory and inhibitory synapses. For example, hippocampal TGF-β1 overexpression or D-serine-mediated TGF-β1 signaling in cortex neurons promoted excitatory synaptogenesis, while TGF-β1 induced inhibitory synaptogenesis through CaM kinase II signaling^11-13^.

Based on the above-addressed information, it is speculated that hippocampal astrocyte-derived factors are important for structural plasticity associated with memory processes. However, the modulatory role of irisin in the dendritic remodeling of hippocampal neurons and astrocyte-secreted factors profile involved in synaptogenesis remains eluive. Herein, we assessed the cytoarchitecture and several astrocyte-derived factors mRNA in hippocampus of subacute irisin-treated mice.

## METHODS

### Experimental mice

Male 7-week-old C57BL/6 mice were obtained from Daehan Biolink, Co., Ltd. (Eumsung, Chungbuk, Korea), and the Animal Care and Use Committee of the Medical School of Ewha Women’s University approved all of the experimental procedures involving animals.

### Golgi staining, neuronal reconstruction, and morphometric analyses

Golgi-Cox staining of brain tissue was performed using a NovaUltra^TM^ Golgi-Cox Stain Kit (IHC World, Woodstock, MD, USA) according to the procedure suggested by the manufacturer. The protocols for Golgi staining, reconstruction, and morphometric analysis have been described in a previous publication by our group^14^.

### Quantitative reverse transcription polymerase chain reaction

The total RNA from mouse brain was extracted, and complementary DNA (cDNA) was synthesized using MultiScribe Reverse Transcriptase (Applied Biosystems, Foster city, CA, USA). Real-time qRT-PCR with SYBR Green PCR Master Mix (Thermo Fisher Scientific, MA, USA) was performed using an ABI 7900 Sequence Detector System (Applied Biosystems). Fold change was calculated using the 2-ΔΔCT method. The primer sequences were as follows: *Pgc1a*: forward 5’-ATG TGA CTG GGG ACT GTA AGA-3’, reverse 5’-GCA GCA CAC TCT ATG TCA CTC-3’; *Fndc5*: forward 5’-ATG AAG GAG ATG GGG AGG AA-3’, reverse 5’-GCG GCA GAA GAG AGC TAT AAC A-3’; *Bdnf*: forward 5’-CAG GAC AGC AAA GCC ACA AT-3’, reverse 5-GCC TTC ATG CAA CCG AAG TA-3’; *Tsp-1*; forward 5′-TGG CCA GCG TTG CCA-3′, reverse 5′-TCT GCA GCA CCC CCT GAA-3′; *Tsp-2* forward 5′-GCA TAG GGC CAA GAG CTT CTG-3′, reverse 5′-CCG GTT AAT GTT GCT GAT GCT-3′; *Sparc* forward 5’-CTGGACGAGAGCAACACC-3’, reverse 5’-AAGAAGTGGCAGGAAGAGTC-3’; Hevin: forward 5′-GGCTGAAGAAAGCCAGACAC-3′, reverse 5′-GTGTCCTTCTGGTTGCCAAT-3′; Tgf-b1: forward 5’-ATTCCTGGCGTTACCTTGG-3’, reverse 5’-CCTGTATTCCGTCTCCTTGG-3’; Gapdh: forward 5’-AACTCCCACTCTTCCACCTTCG-3’, reverse 5’-TCCACCACCCTGTTGCTGTAG-3’

### Statistical analysis

Significant differences between groups were determined using independent t-tests (SPSS for Windows, version 18.0, Chicago, IL, USA). All values are reported as mean ± standard error of mean (SEM). Values of p < 0.05 were considered statistically significant.

## RESULTS

### The seven consecutive days of irisin treatment caused dendritic remodeling in CA1 and CA3, but not dentate gyrus (DG)

**Figure 1.**
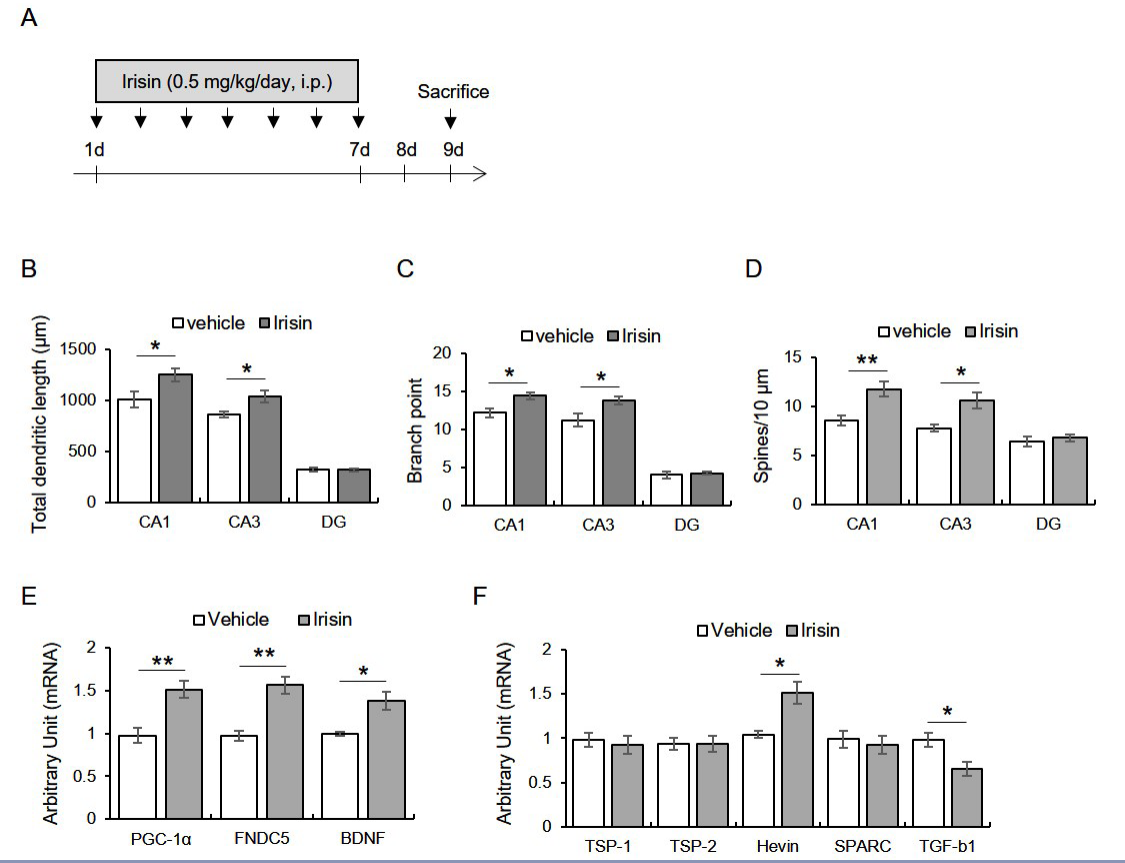
Subacute treatment with irisin promotes dendritic enlargement of hippocampus through enhancing hippocampal transcripts of pgc-1a, fndc5, bdnf, and hevin, but reducing tgf-b1. A. Experimental design. Mice were intraperitoneally injected with irisin for seven days, followed by the sacrifice two days later. B-D. Quantitative analysis of dendritic length, branch point, and spine numbers. E. Quantitative analysis of *pgc-1a*, *fndc5*, *bdnf* mRNA. F. Quantitative analysis of *tsp-1*, *tsp-2*, *hevin*, *sparc*, *tgf-b1*. Data are presented as the mean ± standard error of the mean. *p < 0.05, **p < 0.01.

Mice were intraperitoneally injected with irisin (0.5 μg/kg/day; Phoenix Pharmaceuticals Inc., CA, USA) for seven days, and then morphometry was conducted 2 days later (Fig. 1A). We found that total dendritic length (CA1: t_8_ = -2.38, *p* < 0.05; CA3: t_8_ = -2.59, *p* < 0.05; DG: t8 = 0.16, *p* > 0.05), branch point (CA1: t8 = -2.84, *p* < 0.05; CA3: t8 = -2.50, *p* < 0.05; DG: t_8_ = -0.40, *p* > 0.05), and spine density (CA1: t_8_ = -3.37, *p* < 0.05; CA3: t_8_ = -3.13, *p* < 0.05; DG: t_8_= -0.60, *p* > 0.05) were significantly enhanced by irisin treatment in hippocampal CA1 and CA3 areas, but not DG (Fig. 1B-D).

### The seven consecutive days of irisin treatment upregulated hippocampal PGC-1α, irisin, BDNF, and Hevin transcripts and downregulated TGF-β1

Next, we found that the exogenous irisin treatment enhanced the hippocampal PGC-1α (t_6_ = -3.99, *p* < 0.01), FNDC5 (t_6_ = -5.13, *p* < 0.01), and BDNF (t_6_ = -3.60, *p* < 0.05) mRNA levels (Fig. 1E). Moreover, the hevin (t_6_ = -3.66, *p* < 0.05) mRNA levels were enhanced by irisin treatment, while those of TGF-β1 were significantly reduced by irisin (Fig. 1F). TSP-1 (t_6_ - 0.45, *p* > 0.05), TSP-2 (t_6_ = 0.20, *p* > 0.05), and the SPARC (t_6_ = 0.48, *p* > 0.05) levels were not different between the groups (Fig. 1F).

## DISCUSSION

The current study demonstrated that the subacute treatment with irisin enlarged dendrites of hippocampal CA1 and CA3 neurons. Furthermore, the intraperitoneal treatment with irisin caused the induction of PGC-1α, BDNF, and FNDC5 mRNA, along with the up-regulation of hevin and down-regulation of TGF-β1 transcripts in the hippocampus.

FNDC5/irisin is an exercise-induced myokine under the control of PGC-1α, which induces hippocampal BDNF^1^. The PGC-1α/FNDC5/BDNF signal cascade is recently considered as a key mechanism underlying the exercise-elicited synaptic plasticity, neuroprotection, and cognitive improvement^2,3^.

In the present work, the recombinant irisin treatment for seven days enhanced dendritic length, dendritic arbor, and spine density of hippocampal CA1 and CA3 area, but not the DG area. This result suggests that the exogenous irisin may cross the BBB and affect the structural plasticity of hippocampal neurons. Moreover, this result implied the structural plasticity-promoting effects of FNDC5/irisin. Since the morphological characteristics of dendrites contributes to the processing of neuronal activity and circuitry through synaptic connectivity, which the primary sites of information input between neurons, the structural plasticity-promoting role of FNDC5/irisin contributes to the enhanced synaptic plasticity associated with cognition. This interpretation has some supportive evidence that the peripheral adenoviral-mediated FNDC5 improved hippocampal synaptic plasticity and memory impairment in the AD-model mouse^2,3^. Unlike CA fields, the unchanged cytoarchitecture in the DG area may be attributed to the predominant contribution of irisin to the neurogenic activity, although it was unclear and remains to be investigated further.

We confirmed that the peripheral irisin could induce the hippocampal induction of PGC-1α/FNDC5/BDNF transcripts. This result was consistent with results from the previous studies^2,3,15^, and this implied the coincidence of the peripheral-central PGC-1α/FNDC5/BDNF pathway.

Astrocytes regulate the formation of diverse types of synapses such as glutamatergic^7,8,16^. Additionally, the neuroprotective effect of irisin was not observed in the neuronal culture with the direct treatment with irisin against the Aβ peptide toxicity, but this effect occurred in conditioned media from astrocyte culture with irisin^3^. A study demonstrated that irisin improved astrocytic glucose metabolism and transporter translocation into the membrane^17^. Based on the previous information, the probability that irisin may act through astrocytes attempted us to explore the expression of astrocyte-secreted factors. Notably, hevin mRNA levels were up- and TGF-β1 were down-regulated by irisin treatment. Hevin contributes to the synaptic maturation and formation in the developing brain^18,19^. More recent evidence exhibited hevin-mediated neurite arborization and outgrowth of olfactory ensheathing cells^20^. This previous evidence supported our hypothesis that FNDC5/irisin-facilitated astrocytic secretion of hevin promoted hippocampal dendritogenesis. Studies demonstrated that irisin increased ATP production and energy metabolism in mice carrying muscle-specific PGC-1α by inhibiting TGF-β1^21^. The recent study showed that irisin inhibits TGF-β1 through nuclear factor erythroid 2-related factor 2 in the heart^22^. These previous findings at least partially support our result that the decrease in astrocytic TGF-β1 by FNDC5/irisin may facilitate dendritic enlargement of the hippocampus.

Taken together, exercise-induced myokine, FNDC5/irisin, may contribute to structural plasticity through elevating hevin and/or reducing TGF-β1 from astrocyte.

## References

[1] Boström P, Wu J, Jedrychowski MP, Korde A, Ye L, Lo JC, Rasbach KA, Boström EA, Choi JH, Long JZ, Kajimura S, Zingaretti MC, Vind BF, Tu H, Cinti S, Højlund K, Gygi SP, Spiegelman BM (2012). A PGC1-α-dependent myokine that drives brown-fat-like development of white fat and thermogenesis. *Nature*.

[2] Wrann CD, White JP, Salogiannnis J, Laznik-Bogoslavski D, Wu J, Ma D, Lin JD, Greenberg ME, Spiegelman BM (2013). Exercise induces hippocampal BDNF through a PGC-1α/FNDC5pathway. *Cell Metab*.

[3] Lourenco MV, Frozza RL, de Freitas GB, Zhang H, Kincheski GC, Ribeiro FC, Gonçalves RA, Clarke JR, Beckman D, Staniszewski A, Berman H, Guerra LA, Forny-Germano L, Meier S, Wilcock DM, de Souza JM, Alves-Leon S, Prado VF, Prado MAM, Abisambra JF, Tovar-Moll F, Mattos P, Arancio O, Ferreira ST, De Felice FG (2019). Exercise-linked FNDC5/irisin rescues synaptic plasticity and memory defects in Alzheimer's models. *Nat Med*.

[4] Banker GA (1980). Trophic interactions between astroglial cells and hippocampal neurons in culture. *Science*.

[5] Christopherson KS, Ullian EM, Stokes CC, Mullowney CE, Hell JW, Agah A, Lawler J, Mosher DF, Bornstein P, Barres BA (2005). Thrombospondins are astrocyte-secreted proteins that promote CNS synaptogenesis. *Cell*.

[6] Kucukdereli H, Allen NJ, Lee AT, Feng A, Ozlu MI, Conatser LM, Chakraborty C, Workman G, Weaver M, Sage EH, Barres BA, Eroglu C (2011). Control of excitatory CNS synaptogenesis by astrocyte-secreted proteins Hevin and SPARC. *Proc Natl Acad Sci U S A*.

[7] Allen NJ (2014). Astrocyte regulation of synaptic behavior. *Annu Rev Cell Dev Biol*.

[8] Chung WS, Allen NJ, Eroglu C (2015). Astrocytes Control Synapse Formation, Function, and Elimination. *Cold Spring Harb Perspect Biol*.

[9] Singh SK, Stogsdill JA, Pulimood NS, Dingsdale H, Kim YH, Pilaz LJ, Kim IH, Manhaes AC, Rodrigues WS Jr, Pamukcu A, Enustun E, Ertuz Z, Scheiffele P, Soderling SH, Silver DL, Ji RR, Medina AE, Eroglu C (2016). Astrocytes Assemble Thalamocortical Synapses by Bridging NRX1α and NL1 via Hevin. *Cell*.

[10] Campolongo M, Benedetti L, Podhajcer OL, Pitossi F, Depino AM (2012). Hippocampal SPARC regulates depression-related behavior. *Genes Brain Behav*.

[11] Diniz LP, Almeida JC, Tortelli V, Vargas Lopes C, Setti-Perdigão P, Stipursky J, Kahn SA, Romão LF, de Miranda J, Alves-Leon SV, de Souza JM, Castro NG, Panizzutti R, Gomes FC (2012). Astrocyte-induced synaptogenesis is mediated by transforming growth factor β signaling through modulation of D-serine levels in cerebral cortex neurons. *J Biol Chem*.

[12] Bae JJ, Xiang YY, Martinez-Canabal A, Frankland PW, Yang BB, Lu WY (2011). Increased transforming growth factor-β1 modulates glutamate receptor expression in the hippocampus. *Int J Physiol Pathophysiol Pharmacol*.

[13] Diniz LP, Tortelli V, Garcia MN, Araújo AP, Melo HM, Silva GS, Felice FG, Alves-Leon SV, Souza JM, Romão LF, Castro NG, Gomes FC (2014). Astrocyte transforming growth factor beta 1 promotes inhibitory synapse formation via CaM kinase II signaling. *Glia*.

[14] Leem YH, Park JS, Chang H, Park J, Kim HS (2019). Exercise Prevents Memory Consolidation Defects Via Enhancing Prolactin Responsiveness of CA1 Neurons in Mice Under Chronic Stress. *Mol Neurobiol*.

[15] Choi SH, Bylykbashi E, Chatila ZK, Lee SW, Pulli B, Clemenson GD, Kim E, Rompala A, Oram MK, Asselin C, Aronson J, Zhang C, Miller SJ, Lesinski A, Chen JW, Kim DY, van Praag H, Spiegelman BM, Gage FH, Tanzi RE (2018). Combined adult neurogenesis and BDNF mimic exercise effects on cognition in an Alzheimer's mouse model. *Science*.

[16] Bélanger M, Allaman I, Magistretti PJ (2011). Brainenergy metabolism: focus on astrocyte-neuron metabolic cooperation. *Cell Metab*.

[17] Wang S, Pan J (2016). Irisin ameliorates depressive-like behaviors in rats by regulating energy metabolism. *Biochem Biophys Res Commun*.

[18] Lively S, Brown IR (2008). The extracellular matrix protein SC1/hevin localizes to excitatory synapses following status epilepticus in the rat lithium-pilocarpine seizure model. *J Neurosci Res*.

[19] Lively S, Ringuette MJ, Brown IR (2007). Localization of the extracellular matrix protein SC1 to synapses in the adult rat brain. *Neurochem Res*.

[20] Ge L, Zhuo Y, Wu P, Liu Y, Qi L, Teng X, Duan D, Chen P, Lu M (2019). Olfactory ensheathing cells facilitate neurite sprouting and outgrowth by secreting high levels of hevin. *J Chem Neuroanat*.

[21] Peng H, Wang Q, Lou T, Qin J, Jung S, Shetty V, Li F, Wang Y, Feng XH, Mitch WE, Graham BH, Hu Z (2017). Myokine mediated muscle-kidney crosstalk suppresses metabolic reprogramming and fibrosis in damaged kidneys. *Nat Commun*.

[22] Chen RR, Fan XH, Chen G, Zeng GW, Xue YG, Liu XT, Wang CY (2019). Irisin attenuates angiotensin II-induced cardiac fibrosis via Nrf2 mediated inhibition of ROS/ TGFβ1/Smad2/3 signaling axis. *Chem Biol Interact*.

